# Risk Management in Magnetic Resonance: Failure Mode, Effects, and Criticality Analysis

**DOI:** 10.1155/2013/763186

**Published:** 2013-09-19

**Authors:** Antonella Petrillo, Roberta Fusco, Vincenza Granata, Salvatore Filice, Nicola Raiano, Daniela Maria Amato, Maria Zirpoli, Alessandro di Finizio, Mario Sansone, Anna Russo, Eugenio Maria Covelli, Tonino Pedicini, Maria Triassi

**Affiliations:** ^1^Division of Radiology, Department of Diagnostic Imaging, Radiant and Metabolic Therapy, “Istituto Nazionale Tumori Fondazione G. Pascale,” IRCCS, Via Mariano Semmola, 80131 Naples, Italy; ^2^Department of Electrical Engineering and Information Technologies, University of “Federico II,” Via Claudio, 80125 Naples, Italy; ^3^Department of Preventive Medical Sciences, University of “Federico II,” Via Pansini, 80131 Naples, Italy; ^4^Department of Diagnostic Imaging, Sant'Anna e San Sebastiano Hospital of Caserta, Via Gennaro Tescione, Caserta, Italy; ^5^General Director, “Istituto Nazionale Tumori Fondazione G. Pascale,” IRCCS, Via Mariano Semmola, 80131 Naples, Italy

## Abstract

The aim of the study was to perform a risk management procedure in “Magnetic Resonance Examination” process in order to identify the critical phases and sources of radiological errors and to identify potential improvement projects including procedures, tests, and checks to reduce the error occurrence risk. In this study we used the proactive analysis “Failure Mode Effects Criticality Analysis,” a qualitative and quantitative risk management procedure; has calculated Priority Risk Index (PRI) for each activity of the process; have identified, on the PRI basis, the most critical activities and, for them, have defined improvement projects; and have recalculated the PRI after implementation of improvement projects for each activity. Time stop and audits are performed in order to control the new procedures. The results showed that the most critical tasks of “Magnetic Resonance Examination” process were the reception of the patient, the patient schedule drafting, the closing examination, and the organization of activities. Four improvement projects have been defined and executed. PRI evaluation after improvement projects implementation has shown that the risk decreased significantly following the implementation of procedures and controls defined in improvement projects, resulting in a reduction of the PRI between 43% and 100%.

## 1. Introduction

The prevention of adverse events in health care is one of the elements that constitute the *Clinical Governance Policy* that means, operatively, the implementation of the risk management program as the collection of various actions taken to improve the quality of health care and ensure patient safety, security based on learning from error [1–9].

The clinical risk management is a comprehensive program for the prevention of clinical risk management understood as the clinical, diagnostic, therapeutic, or rehabilitative error probability. The clinical risk management or risk management in radiology regards the system of guidelines, protocols, routes, procedures, and organizational measures to reduce the likelihood of events and potential actions to produce adverse effects or unexpected effects on the health of professionals and/or patients [1–9].

The analysis of processes allows us to take preventive tools for possible faults identification and the definition of improvement actions to optimize work and to minimize the risk of errors for the patient. A careful analysis must aim to identify risks related to the management of all phases of a process of radiological diagnosis, for measuring and setting actions for prevention and control [8–11].

Two methodologies can be used to analyze clinical risk: proactive analysis, which aims to identify and eliminate the criticality of the system before the incident occurs, is based on the analysis of the processes that constitute the activity; it identifies the critical points with the goal of designing secure systems [10, 11]. Reactive analysis provides a study of postaccident, and it is aimed to identify the causes that have allowed the occurrence of the event, in order to reduce future incidents. This method proceeds back against the occurrence of the events: starting from the mistakes of the system of searching for the root causes [9, 10]. Among the techniques of proactive risk analysis we consider the Failure Mode and Effect Analysis (FMEA), qualitative technique that aims at prospectively determining possible failures and their effects on the stability of the entire system, with the aim of redesigning the process itself, or Failure Mode Effects Criticality Analysis (FMECA) that adds to FMEA a quantitative analysis for estimating how critical the identified failures are, with the allocation of an index for facilitating consistent decision making [10, 11]. FMEA and FMECA are strategies developed for identifying the potential errors of a product/process, evaluating the associated risk and assigning a value in terms of importance. The aim is to introduce corrective actions for tackling the more severe problems [1, 2]. 

In radiology, this analysis aims to identify risks linked to the diagnostic process, in particular, to provide information for their evaluation illustrating methods and instruments for the description of organizational processes capable of preventing risks linked to the diagnostic process, and lastly to guarantee elements for effective risk management by adopting improvements [9–11].

The objective of the study is to apply the FMECA proactive analysis for risk management in “Magnetic Resonance Examination” process in order to identify the critical phases (activities) with higher Priority Risk Index (PRI) and to identify possible improvement projects. In order to determine the PRI, three characteristics are needed: probability (probability of the event occurring), severity (severity of the event), and detection (possibility of detecting critical aspects or identifying the failure through controls before the event has produced its negative effects) [9].

The process “Magnetic Resonance Examination” has been performed in Radiology Division of Diagnostic Imaging, Radiant, and Metabolic Therapy Department, “Istituto Nazionale Tumori Fondazione G. Pascale ”—IRCCS, Naples, Italy. FMECA was performed including a qualitative analysis and a quantitative analysis with PRI measure and identifying the most critical phases (activities) of the process, which have defined improvement projects to reduce the clinical risk. PRI evaluation was performed after improvement projects implementation to evaluate the effectiveness of the strategies applied to “Magnetic Resonance Examination” process.

## 2. Material and Methods

### 2.1. Proactive Analysis FMECA

The FMECA analysis, as required by the Joint Commission on Accreditation Standard of Healthcare Organizations (JCAHO) [10, 11], involves the following steps:

1. decomposition of the process, product, or system into subsystems (phase investigation);
2. individuation of potential “failure mode”;
3. individuation of the possible effects for each “failure mode”;
4. implementation of a root cause analysis for the most critical effects;
5. process redesign to minimize the clinical risk;
6. testing and implementation of redesigned process;
7. implementation of a strategy to preserve the results in long term.

Proactive analysis provides both qualitative and quantitative analysis. In the qualitative analysis all the possible types of error/failure, the potential causes and their effects were listed. In the quantitative analysis for each considered element has been associate a judgment on predefined criteria, built on a scale from 1 to 10, in order to calculate a Priority Risk Index (PRI).

PRI is calculated as the product of three characteristics: P, probability of the event occurring (Table 1); S, severity of the event (Table 2); D, error detection (Table 3). It can take a maximum value of 1000 (10 × 10 × 10: product of the maximum scores) and a minimum value of 1 (1 × 1 × 1: product of the minimum scores).

The FMECA analysis was performed by a multidisciplinary team with unanimous consensus in assigning scores to PRI calculation. PRIs obtained were sorted in descending order; the critical threshold for each PRI has been chosen using the Pareto criterion: a value >200 corresponds to a high risk, a value >100 corresponds to a medium risk, a value >50 corresponds to a low risk, and a value <50 corresponds to a very low risk [8].

### 2.2. Magnetic Resonance Devices

In this study we consider the activity and organization of two magnetic resonance (MR) devices.

1. Siemens Symphony 1.5 T MRI.
2. AURORA 1.5 T dedicated breast MRI.

The service of “Magnetic Resonance Examination” is addressed to included inpatients, outpatients, and preadmission patients.

### 2.3. Job Group

FMECA analysis was performed by a multidisciplinary group including:

1. administrative,
2. professional nurses,
3. radiographers,
4. radiologists, and
5. biomedical engineers.

The working group consisted of 2 administrative, 5 professional nurses, 4 radiographers, 3 radiologists, and 1 biomedical engineer.

The FMECA analysis was performed with periodic meetings (2 per month for six months).

Control of improvement procedures implementation has been made by means of audit and time stop procedures by the Director of Radiology Division, as being medically responsible of magnetic resonance equipment.

## 3. Results

### 3.1. Investigation Phase

Magnetic Resonance Examination process was divided into 6 phases for a total of 28 activities:

1. exam reservation (divided into 2 activities);
2. activity organization (divided into 6 activities);
3. patient reception and preparation (divided into 4 activities);
4. diagnostic session preparation (divided into 5 activities);
5. exam execution (divided into 4 activities);
6. closing examination (divided into 7 activities).

For each phase have been defined in detail:

1. who normally performs the action;
2. how the action is performed.

### 3.2. Quantitative and Qualitative Analysis

For each of 28 activities all potential defects and consequences (qualitative analysis) have been identified. More defects were detected for a single task, for a total of 31 errors (2 for the patient reception, 3 for storing exam). Each potential defect was attributed a score according to probability, severity, and detection scales. PRIs were calculated and were listed in descending order (quantitative analysis).

Table 4 shows all tasks with PRI ≥100, as well as the PRI for the phase “Activities Organization” for which it was considered appropriate to report an improvement project.

### 3.3. Improvement Projects

In this study we have identified four improvement projects, for the activities with higher PRIs:

1. patient reception,
2. patient schedule compilation,
3. closing exam, and
4. organization of activities with routine inspections.

In the following the improvement projects for the four activities were reported. Each improvement project provides a new procedure, a period of training, and a control phase using time stop and audit.

#### 3.3.1. Improvement Project “Patient Reception”

It is as follows.

 (1) Procedure includes
  - receptionist delivering to each patient an examination label underlining the ID number;
  - nurse/radiographer verifing the patient arrival order by referring to the work list where the patients are listed in order of acceptance. The work list is visible on dedicated PC in MR area;
  - staff who introduces the patient in MRI diagnostics calls the patient with the ID number.
 (2) Staff training.
 (3) Time stop and audit each 15, 30, and 45 days.

#### 3.3.2. Improvement Project “Patient Schedule Compilation”

It is as follows.

1. Procedure includes
  - reading of the information schedule and explanations on the examination procedure by Medical Doctor/Nurse/Radiographers;
  - compilation of patient schedule by Medical Doctor;
  - collection of informed consensus signed by patient.
2. Staff training.
3. Audit sample on completed schedule.
4. Time stop and audit each 15, 30, and 45 days.

#### 3.3.3. Improvement Project “Closing Exam”

It is as follows.

1. Procedure includes
  - verification of the closure examination on RIS (Radiology Information System), responsibility of the radiographers;
  - verification of the storing exams on DVD, responsibility of the radiographers;
  - verification that work list corresponds to exams list sent to PACS (Picture Archiving and Communication System), responsibility of the radiographers.
2. Staff training.
3. Audit sample.

#### 3.3.4. Improvement Project “Organization of Activities with Routine Inspections”

Although is among the most significant in this study, an improvement project for this activity, has been reported which includes

1. drafting check list, differentiated for nurse and radiographers, about routine inspections;
2. staff training about duties and responsibilities;
3. staff training about check list use (check list is personal for each operator, to be filled daily and delivered monthly with date and signature);
4. check list storage;
5. audit sample.

### 3.4. PRIs Evaluation after Improvement Projects Implementation

Risk Analysis results consist of PRIs evaluation after improvement projects implementation for the four phases (about six months after the end of proactive analysis): patient reception, patient schedule compilation, closing examination, and organization of activities.

Table 5 shows the PRIs recalculated for the four stages. For patient reception the PRI was reduced by 99.6%; for patient schedule compilation the PRI was reduced by 42.9%; for closing examination PRI was reduced by 100%, and for organization of activities the PRI was reduced by 75%.

## 4. Discussions and Conclusions

The objective of the study is to apply the FMECA proactive analysis for risk management in “Magnetic Resonance Examination” process in order to identify the critical phases (activities) with higher Priority Risk Index and to identify possible improvement projects to reduce clinical risk associated with error occurrence. 

In the literature only a precedent similar study was individuated; Centonze et al. [8], with the aim of providing a clearer understanding of the tools used for evaluating risk in the radiological setting, perform a proactive analysis applied to CT, and a reactive analysis was performed following a sentinel event triggered by a CT study allocated to the wrong patient in the RIS-PACS system. Centonze et al. [8] conclude that the reactive analysis of the principal critical radiological elements that contributed to the sentinel event emphasizes the pressing need to change the management of CT healthcare services about RIS-PACS system.

In this study, the results showed that the most critical stages were the patient reception phase, the stage of patient schedule completion, the phase of closing examination, and the organization of activities.

A revaluation of PRIs was performed after improvement projects implementation, to evaluate the effectiveness of the applied strategies.

PRIs evaluation after improvement project implementation showed that the risk associated with these phases decreased significantly following the application of procedures and controls defined in improvement projects, resulting in a reduction of the PRIs between 43% and 100%. Therefore, the use of proactive analysis, significantly reduced the clinical risk of the process “Magnetic Resonance Examination” proving highly effective.

## Figures and Tables

**Table 1:** Risk estimation: probability of the event occurring and relative score system.

Probability score system		
Probability	Percentage value	Score
Remote	0	1
Low	1‰–5‰	2-3
Moderate	5‰–1%	4–6
High	1%–5%	7-8
Very high	<50%	9-10

**Table 2:** Risk estimation: severity of the event and relative score system.

Severity score system		
Severity	Severity criterion	Score
Extremely dangerous	Cause to death	10
Dangerous	Injury or chronic disabilities	9
Very high	Extension of hospitalization with outcomes at discharge	8
High	Extension of hospitalization without outcomes at discharge	7
Moderate	Damage that requires treatment with more drugs	6
Low	Damage that requires treatment with minor drugs	5
Very low	Damage that requires observation and diagnostic procedures	4
Less	Minor damage that does not require treatment	3
Minimum	Negligible damage that does not require treatment	2
Nothing	No result	1

**Table 3:** Risk estimation: detection of the error and relative score system.

Detection score system	
Detection	Score
Nothing	10
Very low	9
Low	7-8
Medium	5-6
High	3-4
Very high	1-2

**Table 4:** Qualitative and quantitative analysis.

Phase	Qualitative analysis				Quantitative analysis			
	Activity	Person in charge	Potential defect	Consequence	P	G	D	PRI
Patient reception and exam preparation	Reception	Nurse	Privacy violation	Complaint	10	7	8	560
Patient reception and exam preparation	Reception	Nurse	Wrong compilation of patient schedule	Inadequate history patient	7	5	8	280
Closing exam	Storing exam on DVD	Radiographers	Failure storing and/or loss of DVD	Exam loss	2	10	8	160
Closing exam	Sending exam to PACS	Radiographers	Failure sending exam to PACS	Exam loss	2	10	7	140
Closing exam	Closing exam on RIS	Radiographer/Medical Doctor	Failure verification of patient data correctness	Statistic error	7	2	9	126
Activities organization	Routine inspections	Nurse/Radiographers	Inadvertency of a control	Malfunction	4	6	2	48

**Table 5:** PRIs evaluation after improvement projects implementation.

Phase	Qualitative analysis		Quantitative analysis			
	Potential defect	Consequence	P	S	D	PRI
Patient reception	Confusion: people do not understand ID number	Delay in patient identification	2	1	1	2
Patient schedule compilation	Distraction from the execution of the exam/reporting	Time examination elongation possible loss of findings accessories	10	2	8	160
Closing exam	Nothing	Nothing	1	1	1	1
Activities organization with routine inspections	For new staff: possible error in the rules while implementing check list	Not accurate inspection	2	2	3	12

## References

[1] Reason J (2002). Combating omission errors through task analysis and good reminders. *Quality and Safety in Health Care*.

[2] Reason JT, Carthey J, de Leval MR (2001). Diagnosing “vulnerable system syndrome”: an essential prerequisite to effective risk management. *Quality in Health Care*.

[3] Bord RJ, O’Connor RE (1992). Determinants of risk perceptions of a hazardous waste site. *Risk Analysis*.

[4] Bradbury J (1989). The policy implications of differing concepts of risk. *Science Technology & Human Values*.

[5] Covello VT, Mumpower J (1985). Risk analysis and risk management: an historical perspective. *Risk Analysis*.

[6] Renn O (1990). Risk perception and risk management: a review. *Risk Abstracts*.

[7] Golfieri R, Pescarini L, Fileni A (2010). Clinical risk management in radiology, part I: general background and types of error and their prevention. *Radiologia Medica*.

[8] Centonze M, Visconti D, Doratiotto S (2010). Clinical risk management in radiology, part II: applied examples and concluding remarks. *Radiologia Medica*.

[9] Caroll R (2012). *Risk Management Handbook for Health Care Organizations*.

[10] Borgovini R, Pemberton S, Rossi M (1993). *Failure Mode, Effects and Criticality Analysis (FMECA)*.

[11] Sicurezza dei pazienti e gestione del rischio clinico http://www.salute.gov.it/imgs/c_17_pubblicazioni_640_allegato.pdf.

